# Effectiveness of Cardiac Rehabilitation in Myocardial Infarction Patients After Percutaneous Coronary Intervention

**DOI:** 10.7759/cureus.26684

**Published:** 2022-07-09

**Authors:** Zaid A Shah, Qazi Muhammad Jamal, Naveed Ullah, Tufail Ahmad, Moiz Ahmed

**Affiliations:** 1 Department of Cardiology, Khyber Teaching Hospital, Peshawar, PAK; 2 Department of Paediatrics, Qazi Hussain Ahmed Medical Complex, Nowshera, PAK; 3 Department of Cardiac Surgery, Hayatabad Medical Complex, Peshawar, PAK; 4 Department of Medicine, Jinnah Postgraduate Medical Centre, Karachi, PAK

**Keywords:** rehabilitation, myocardial infarction, cardiovascular, angina, acute coronary syndrome

## Abstract

Background

Post myocardial infarction rehabilitation can have a positive impact on overall patient health. Therefore, the present study investigated the role of cardiac rehabilitation in reducing the frequency of arrhythmias, recurrent angina, readmission, and mortality in patients who underwent percutaneous coronary intervention.

Methodology

A prospective observational study was conducted at the Cardiology Department, Khyber Teaching Hospital, Pakistan, between 1^st^ March 2021 and 30^th^ May 2021. All patients who were discharged after being diagnosed with acute myocardial infarction were included in the study. Patients who were not able to give consent, had physical fragility, mental impairment, or those who have critical illness were excluded from the study. 40 patients underwent cardiac rehabilitation while the other 40 acted as controls. The cardiac rehabilitation group patients were asked to engage in 15-30 minutes of activity daily and keep a record of all their activities. Death, recurrence, rhythm abnormalities, rehospitalizations, and BMI (body mass index) were all documented on a predesigned proforma and compared between intervention and control. A three-month-long follow-up plan was established.

Results

A total of 80 patients were enrolled in the study. Post-infarction angina (p = 0.0012) was significantly higher in patients who did not receive cardiac rehabilitation (CR). The incidence of arrhythmias was significantly higher in the control group as compared to the rehab group (p=0.002). Moreover, the mean left ventricular ejection fraction (LVEF) was also significantly higher in patients who underwent CR as compared to the control group (44.76 ± 13.8 vs. 42.9 ± 13.5, p = 0.01). There was no significant difference between the post-intervention BMI in the rehab group.

Conclusion

In conclusion, the present study findings revealed that mild to moderate cardiac rehabilitation (CR) was related to the less frequent occurrence of post-infarction angina and arrhythmias. Moreover, we found that patients who received CR experienced a significant improvement in left ventricular ejection fraction (LVEF) as compared to the control group. However, further large-scale studies from multiple centers are warranted.

## Introduction

Coronary artery disease (CAD) includes a range of presentations from asymptomatic atherosclerosis and stable angina (SA) to acute myocardial infarction (AMI) [1]. AMI is the most severe of these heart diseases and is amongst the most common causes of mortality and morbidity around the world, especially in the geriatric population. With rising life expectancy worldwide, geriatric populations continue to expand. This is cause for concern in light of the deleterious impact of AMI on the lives of these individuals [2]. 

The current standard protocols for the management of AMI involve the frequent use of percutaneous coronary intervention (PCI; Angioplasty with stenting) to restore perfusion. PCI has demonstrated a tremendous reduction in mortality and has allowed for early discharges [3]. Furthermore, it also improved the quality of life in patients with myocardial infarction [4]. Reintegration into society and return to pre-disease health status nevertheless continues to be a public health problem.

In 2019, a study published by Rehman et al. revealed that the standard of clinical practice in Pakistan did not meet the expectations of clinical guidelines, and greater compliance with guidelines resulted in a lengthier inpatient stay [5]. Moreover, the literature reveals that the COVID-19 pandemic had a huge impact on the overall epidemiology of many diseases, including ischemic heart diseases. Noorali et al. revealed a striking decrease in hospitalizations secondary to ACS, necessary diagnostic investigations (i.e., angiography), and interventions (i.e., PCI and coronary artery bypass graft [CABG]) in an already researched and reserved poor nation [6].

To improve the healthcare and social outcomes of patients following an intervention, the development of a rehabilitation program has been an enduring area of research [4]. In the last four decades, cardiac rehabilitation (CR) has come to be considered an essential part of the management of patients suffering from CAD. While studies are abundant globally, in the regional context, there are very few studies from South Asia exploring the mortality and morbidity of cardiac patients adhering to rehabilitation programs along with medical and surgical management [4-8].

CR comprises an outpatient collaborative program that aims to help cardiac patients promptly readjust and improve the physical, psychological, and social aspects of their lives [7]. The objective is to educate and help maintain cardiovascular fitness appropriate for the individual, improve diet and lifestyle, and support mental health. Originally consisting exclusively of exercise, CR now also gives weight to and prioritizes mental health along with promoting professional counseling for mood disorders and stress reduction following a cardiovascular event [8].

CR is presently recommended as per guidelines for a wide range of acute and chronic heart diseases and cardiac interventions such as coronary artery bypass grafting (CABG), acute coronary syndromes (ACS; stable and unstable angina, non-ST-elevation myocardial infarction [NSTEMI], and ST-elevation myocardial infarction [STEMI]), heart failure with reduced ejection fraction (HFrEF), stenting procedures, and valvular surgeries [9]. According to an adamantine collection of past studies and research, CR has been shown to considerably improve health outcomes. It is also an indispensable resource for managing a substantial part of the continuum and a variety of cardiac pathologies [10].

Presently, regardless of the recommendations, referral to CR services by clinicians does not meet the intended benchmark, and patient compliance is low. There is a need for adequate programs to be put in place to increase the number of patients being referred for CR and to improve patient adherence. Due to the scarcity of the subject in the Pakistani population, we conducted the research to study the role of cardiac rehabilitation in patients with a recent history of myocardial infarction. Therefore, the present study investigated the role of cardiac rehabilitation in reducing the frequency of arrhythmias, recurrent angina, readmission, and mortality in patients who underwent percutaneous coronary intervention.

## Materials and methods

A prospective observational study was conducted at the Cardiology Department, Khyber Teaching Hospital, Pakistan, between 1^st^ March 2021 and 30^th^ May 2021. After obtaining ethical approval from the ethical board (# 5022), data acquisition was initiated.

All patients who underwent percutaneous coronary intervention in the last 30 days at the study center following their diagnoses of acute myocardial infarction (AMI) were eligible to partake in the study. The AMI was diagnosed as per the following criteria: angina lasting for more than 30 minutes, electrocardiographic features, and cardiac enzymes (troponin levels of 0.60 ng/mL and higher) after four hours of symptoms presentation.

Patients who were not able to give consent, had physical fragility, mental impairment, or had a critical illness were excluded from the study. All eligible patients were divided into two groups: an intervention and a control group, using a systematic randomized sampling technique. Both groups were followed up for up to three months. During this time, the study group was offered cardiac rehabilitation in addition to the standard treatment, while the control group was given the standard treatment only.

Cardiologists routinely watched over the process of administration of community-based cardiac rehabilitation (CR). Following percutaneous coronary intervention (PCI), the control group was managed according to standard medical practice and received regular care, while the intervention group received CR as part of their standard management plan. There was no difference between the care provided to the two groups except the CR, which was offered to the study group only. CR consisted of aerobic exercises designed by cardiologists. They were customized for participants based on their current health and risk status.

Even though participants were encouraged to come daily for cardiac rehabilitation, it was not practical to travel to the hospital back and forth every day for many patients. Since the majority of these patients belonged to low socioeconomic status, transport expenses were not affordable for many patients. Therefore, considering these very real limitations, cardiologists encouraged the patients to at least visit regularly for one week daily to understand how to exercise.

The most common type of exercise was walking or brisk walking for at least 15- to 30-minutes daily. Participants were encouraged to regain mobility after their procedure as soon as it was tolerable. Additional exercises such as upper and lower extremity movement when supine in bed and gentle walks in the patients’ immediate vicinity were also included in the training protocol. All training that participants partook in was carried out as per the guidelines of the American Heart Association (AHA) [11]. All training was under supervision. Only mild-moderate intensity exercise was recommended. Ten minutes were dedicated to warm-up during which the trainer showed how to stretch and warm the muscles before the exercise. Once the patients were confident that they can do the exercises at home, they were requested to maintain a regular follow-up for physical assessment. Each patient was requested to maintain a log for recording their heart rate and blood pressure using ambulatory blood pressure devices. Moreover, all patients recorded themselves engaging in exercise daily. However, the video recordings were not included in the study analysis. Self-recordings of the patients engaging in exercise ensured that patients were following the doctor’s instructions. Initially, patients were asked to exercise twice daily, but as they developed more strength, the frequency of exercise was increased to three to four times a week. A follow-up visit was maintained every four weeks till three months of the study. Participants were advised to refrain from running amidst exercise. The control group continued the post-discharge treatment as per protocol.

During the follow-up visit, patients' heart rate, blood pressure, frequency of exercise, walking distance, and weight were assessed. Apart from that, in-hospital mortality, three-month all-cause mortality, frequency of arrhythmias, recurrences, and readmissions were also documented. A predefined proforma was used to collect and record data. Arrhythmias were determined by using history, symptoms, and electrocardiography.

Cardiac rehabilitation was labeled as successful if the patient had improved cardiac function at three months of rehabilitation. Cardiac function was evaluated by left ventricular ejection fraction (LVEF) using echocardiography. The LVEF of all patients was assessed twice during the study. At baseline and three months of rehabilitation. Sociodemographic variables, such as gender, age, body mass index, and comorbidities, such as diabetes mellitus, and hypertension, were noted in the proforma for both groups. There was no difference between the sociodemographic features (Table 1) between the two groups. These factors, such as age or gender, could have an impact on the effectiveness of cardiac rehabilitation; therefore, the effectiveness was stratified according to the above-mentioned variables. Weight before and after the rehabilitation was also observed in both groups at baseline (before PCI) and after the study period (three months postoperatively). Weight was measured using an electronic scale during the first and then the follow-up visits.

All statistical analytics were executed with IBM Corp. Released 2015. IBM SPSS Statistics for Windows, Version 23.0. Armonk, NY: IBM Corp. software. Analysis was carried out for both the control and rehabilitation groups separately and in comparison. The continuous data were expressed as mean and standard deviation. The t-test was utilized in the interpretation of measurement data between the control and rehabilitation groups. The Pearson Chi-squared test was utilized to contrast the categorical variables. The paired t-test (for LVEF) or chi-square test (for categorical variables such as mortality, arrhythmias, etc.) were utilized to contrast data of each group before and after the intervention. The p-value at < 0.05 was deemed as statistically significant, with the power of study as 80%. Any p-value of 0.05 or greater meant that no significant difference was observed. 

## Results

A total of 80 patients were enrolled in the study. Forty patients underwent cardiac rehabilitation, while the other 40 acted as controls. The mean age of the patients in the rehab group and control group was 65.22 ± 6.21 years and 68.78 ± 7.019 years, respectively. The majority of the participants were males and had hypertension, as illustrated in Table 1. The two groups were matched with respect to the sociodemographic factors. 

**Table 1:** Baseline differences between the rehabilitation group and the control group LVEF: Left ventricular ejection fraction, BMI: Body mass index, PCI: Percutaneous coronary intervention

Parameter	Rehab group	Control group	p-values
Gender (males %)	34 (85%)	30 (75%)	0.264
Age, years	65.22 ± 6.21	68.78 ± 7.019	0.7051
PCI target			0.922
Left anterior descending artery	18 (45%)	20 (50%)	
Left circumflex	9 (22.5%)	9 (22.5%)	
Right coronary artery	11 (27.5%)	10 (25%)	
Left main artery	2 (5%)	1 (2.5%)	
Comorbidities			
Hypertension	33 (82.5%)	35 (87.5%)	0.531
Diabetes mellitus type 2	15 (37.5%)	17 (42.5%)	0.648
Overweight or Obese (BMI > 29.9 kg/m^2^)	27 (67.5%)	27 (67.5%)	1
Baseline LVEF	29.83 ± 7.9	30.37 ± 7.2	0.7502

Table 2 compares statistics of the outcomes between both the groups. Dissimilarities were seen in the categories of all-cause mortality, re-infarction, and readmission rates, though these were not statistically significant (p > 0.05). Whereas post-infarction angina (p = 0.0012) was significantly higher in patients who did not receive cardiac rehabilitation (CR). The incidence of arrhythmias was significantly higher in the control group as compared to the rehab group (p=0.002). Moreover, the mean left ventricular ejection fraction (LVEF) was also significantly higher in patients who underwent CR as compared to the control group (44.76 ± 13.8 vs. 42.9 ± 13.5, p=0.01). 

**Table 2:** Comparison of the cardiovascular event incidence between the rehabilitation and the control groups LVEF: Left ventricular ejection fraction

Cardiovascular events	Rehab group	Control group	p-value
All cause mortality (within three months)	5 (12.5%)	12 (30%)	0.056
Recurrence	7 (17.5%)	11 (27.5%)	0.284
Arrhythmias	8 (12.5%)	21 (52.5%)	0.002
Readmission	4 (10%)	3 (7.5%)	0.692
Post-infarction angina	8 (20%)	22 (55%)	0.0012
LVEF (after three months)	44.76 ± 13.8	42.9 ± 13.5	0.01

Table 3-5 shows the change in body mass index (BMI) in patients in the rehab group and control group. There was no statistically significant difference between the rehab versus the control group (Table 3). 

There was no significant difference between the post-intervention BMI in the rehab group (Table 4). 

**Table 3:** Pre-intervention and post-intervention body mass index (BMI) comparison in rehabilitation group versus the control group BMI: body mass index

Pre-intervention BMI	Rehab group	Control group	p-value
Normal Weight	13 (32.5%)	12 (30%)	0.81
Overweight	18 (45%)	19 (47.5%)	0.822
Obese	9 (22.5%)	8 (20%)	0.785
Post-intervention BMI	Rehab group	Control group	p-value
Normal Weight	16 (40%)	13 (32.5%)	0.485
Overweight	16 (40%)	20 (50%)	0.369
Obese	8 (20%)	7 (17.5%)	0.775

**Table 4:** Pre-intervention and post-intervention body mass index (BMI) comparison in rehabilitation group

Body Mass Index (BMI)	Pre-intervention	Post-intervention	p-value
Normal Weight	13 (32.5%)	16 (40%)	0.485
Overweight	18 (45%)	16 (40%)	0.651
Obese	9 (22.5%)	8 (20%)	0.785

There was no significant difference between the post-intervention BMI in the control group (Table 5). 

**Table 5:** Pre-intervention and post-intervention body mass index (BMI) comparison in the control group

Body Mass Index (BMI)	Pre-intervention	Post-intervention	p-value
Normal Weight	12 (30%)	13 (32.5%)	0.81
Overweight	19 (47.5%)	20 (50%)	0.823
Obese	8 (20%)	7 (17.5%)	0.766

## Discussion

The newest guidelines with regards to cardiac rehabilitation (CR) reference a reduction in mortality due to all causes by 20%. Kabboul et al. undertook a systematic review and meta-analysis of previously published randomized control trials regarding the various aspects of CR [9]. Current evidence reaffirmed the merit of CR in decreasing mortality and morbidity when followed thoroughly and meticulously [11-13].

The current standard of clinical care for acute myocardial infarction (AMI) is very methodical, refined and strictly upheld. Regardless, referrals, emphasis, and compliance to CR still disproportionately lag behind the medical management of AMI. Research regarding community-based CR is seldom reported. After five decades of exploration and advancement regarding the role of CR in mortality reduction, clinical research now substantiates that CR is effective. This effectiveness was demonstrated in meta-analyses that established that exercise-based CR reduces all-cause mortality and reinfarction after the initial AMI [14-17]. Aside from mortality, CR also notably bettered the quality of life, decreased the occurrence of cardiovascular incidents, and markedly enhanced maximal oxygen consumption and, thus, physiologic functioning. Post-rehabilitation mortality was correlated inversely with the duration of the CR [18,19].

In this study, we found that patients who underwent exercise-based rehabilitation programs were less likely to experience post-infarction angina and arrhythmias. Moreover, we found that patients who received CR experienced a significant improvement in left ventricular ejection fraction (LVEF) as compared to the control group. Zhang et al. have also produced the same results recently. They demonstrated that patients undergoing a thorough rehabilitation program, i.e., the rehabilitation group had a statistically significant reduction in both post-infarction angina and re-hospitalization rates with a p-value of <0.01 [20].

According to a meta-analysis exploring the impact of exercise on LVEF after AMI revealed that when the exercise program begins sooner after a MI and is continued for more than three months, it has the greatest positive impact on LVEF in medically stable post-MI patients [21]. Another study published by Bourkhris et al. found that the individuals with coronary artery disease who underwent cardiac rehabilitation had improvements in most of the ventricular repolarization indicators and had reduced occurrence of ventricular arrhythmias [22]. This also supports the conclusion of this study.

At present, guidelines regarding the treatment and management of adverse cardiac events such as ACS and AMI identify post-intervention CR programs as essential. As per the majority of clinical guidelines for patients suffering from heart failure with reduced EF (HFrEF) and coronary artery disease (including ACS and AMI), exercise-based CR is a Class IA indication. Regardless of the vast and growing body of evidence, and numerous guidelines supporting the role of CR in this group of patients, the desired benchmark has not been reached. The literature proclaims that both inpatient and outpatient CR programs globally do not produce outstanding outcomes. The attendance and adherence to both forms of CR differs geographically but nonetheless, as stated results are frequently disappointing. This dilemma has been discussed, and the proposed rationales primarily consist of a lack of referral to CR programs by the treating clinician and disfavor for CR among clinicians due to perceived lack of safety. Rauch et al. clearly counter this notion held by many clinicians worldwide regarding the safety of CR for AMI patients by demonstrating a notable lowering of one-year mortality in these patients with early CR under supervision and guidance [23].

One of the limitations we faced during our study was that many patients could not come to the hospital for supervised rehabilitation, so we had to train these patients as well as their attendants so that they can do these simple exercises regularly at home. The attendants were trained to document blood pressure and heart rate of the patients. Second limitation of the study was that the sample size for this study was relatively limited, consisting of 80 participants enrolled from one center. Our findings justify the requirement for additional studies for patients post-AMI with a greater number of participants and extended CR programs. These larger-scale studies may prove to be more consequential in further establishing the importance and effect of CR in this setting and improving physician practice and compliance of CR.

## Conclusions

In conclusion, the present study findings revealed that mild to moderate cardiac rehabilitation (CR) was related to the less frequent occurrence of post-infarction angina and arrhythmias. Moreover, we found that patients who received CR experienced a significant improvement in left ventricular ejection fraction (LVEF) as compared to the control group. However, further large-scale studies from multiple centers are warranted.
